# Enhancement of the duckweed biomass and starch production utilizing biogenic MnO and ZnO microparticles

**DOI:** 10.1016/j.btre.2025.e00907

**Published:** 2025-07-16

**Authors:** Maryam Anar, Sajjad Kamal Shuvro, Muhammad Farooq Hussain Munis, Masaaki Morikawa

**Affiliations:** a Department of Plant Sciences, Faculty of Biological Sciences, Quaid-i-Azam University, Islamabad 45320, Pakistan; b Graduate School of Environmental Earth Science, Hokkaido University, N10-W5, Kita-ku, 060-0810 Sapporo, Japan

**Keywords:** Biological microparticles, MnO, ZnO, Duckweed, Plant growth-promoting agent

## Abstract

The effects of biologically synthesized MnO and ZnO microparticles (MPs), were evaluated on the growth of *Lemna minor*. Both MnO and ZnO MPs promoted the growth of L. *minor* at low concentrations of 1–8 mg/L. In contrast, 1000 mg/L MnO and ZnO MPs reduced the growth of L. *minor*. It was also found that the starch content of L. *minor* and *Spirodela polyrhiza* was enhanced upon growing in 1 mg/L of MnO MPs when compared to no MnO MPs. These effects were suggested to be due in part to the fact that biogenic MPs harbored small but significant amounts of inorganic phosphorus. Our findings indicate that biologically synthesized MnO and ZnO MPs can act as novel plant growth regulators to enhance the production of useful duckweed biomass with high starch content.

## Highlights

• Novel function of biogenic MnO and ZnO microparticles (MPs) have been reported in duckweed.
• Biogenic MnO and ZnO were found to incorporate phosphorus from the bacterial cell lysate upon forming MPs.
• MnO and ZnO MPs significantly promoted the duckweed growth and starch production even at 1 mg/L.
• This report provides a new perspective on nanobiotechnology.

## Graphical abstract

**Figure:**
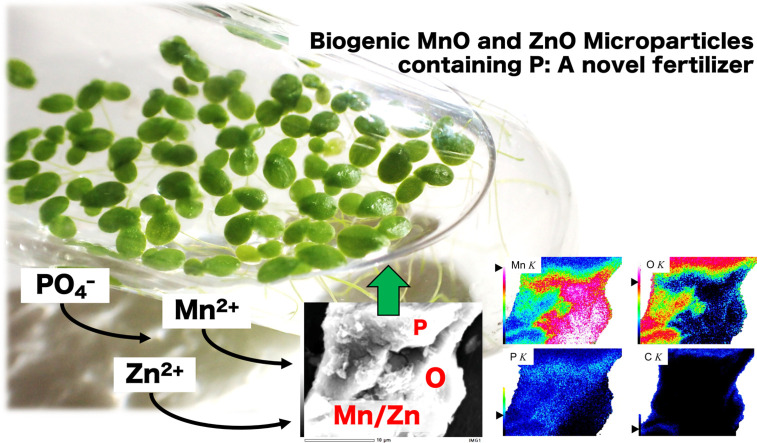

## 1 Introduction

Nanotechnology has been highlighted for its great potential in areas ranging from basic science to industry. Various technologies have been developed to produce nanomaterials with specific properties for various applications [1,2]. Biological nanotechnology is a new field that combines biological materials with chemical and physical operations to create nanoparticles (NPs) [3]. The synthesis of metal oxide NPs using the cell lysates of bacteria, fungi, and plants is inexpensive and can be easily scaled up for commercial production, in addition to eco-friendliness and biocompatibility [[4], [5], [6]]. It has been reported that the cell lysates can ultimately oxidize metal ions under aerobic conditions to form and stabilize metal oxide NPs [7].

The properties of NPs closely correlate to their size and shape; thus, controlling the size plays important role in conferring desired attributes to the resulting particles [8]. However, NPs, ranging from 1 to 100 nm in size, and larger microparticles, MPs, are not often separately described about their different functions. In addition to applications in medicine and electronics, mixture of NPs/MPs have also shown their potential in agriculture, particularly for enhancing plant growth and conferring resistance to environmental stresses [9,10]. In this study, we have selectively prepared metal oxide MPs, which have not yet received much attention on their specific function. MPs are defined as particles with a size larger than several hundred nm.

Duckweed is an aquatic floating plant of the Lemnaceae family, which is classified into five genera: *Spirodela, Landoltia, Lemna, Wolfiella* and *Wolffia*. Duckweed generally reproduces asexually without flowering, so that it can maximally double its biomass in a couple of days, enabling greater productivity with less labor of harvest as compared to soil crops. Each hectare of duckweed pond under favorable conditions is estimated to yield 55 tons of dry matter annually [11]. The duckweed biomass has been highlighted for its multifunctionality due to its high protein and starch contents with trace amount recalcitrant lignins [[12], [13], [14]]. Moreover*,* duckweed is also capable of removing pollutants from environmental waters [15,16].

This study has focused on the biological synthesis of MnO and ZnO MPs using bacterial cell lysates and examined their effects on the duckweed. The findings contribute to the technology development for enhancing plant growth, while also to highlight duckweed as a potential target material in the application of metal MPs.

## 2 Materials and methods

### 2.1 Synthesis of biogenic MnO and ZnO NPs/MPs

NPs/MPs were synthesized as described by Sinha et al [17]. *Bacillus subtilis* 168 was cultured in Luria Bertani (LB) medium at 35–37 °C for 24 h. When the culture had an OD_600_ ∼ 1.0, it was centrifuged to remove medium, and approximately 10 g of fresh bacterial cells was suspended within 150 mL distilled water and further incubated for a week at 45 °C in an orbital shaking incubator. After one week of incubation with the bacterial cell suspension, the cells and debris were removed by centrifugation. The resulting clear cell lysates were filter-sterilized using 0.22 μm Millex GV (Merck KGaA, Darmstadt, Germany) membrane filters and used to prepare NPs/MPs as detailed below.

Bacterial cell lysates and 10 mM MnSO_4_ or ZnSO_4_ solutions were mixed in a 1:1 ratio in a beaker and gently agitated for 24–48 h at room temperature. The synthesis of NPs/MPs was monitored by observing changes to the color of the mixture. When the color changed from yellowish to brownish, the sample was centrifuged at 5800 × *g* for 20 min. The resultant pellets containing MPs were washed with pure water to remove NPs and impurities and dried overnight at 60 °C [10]. The resulting MnO and ZnO MPs were resuspended in pure water, dispersed by sonication at 100 V, 40 kHz for 30 min (Sonifier 250; Branson Ultrasonics Corp., Danbury, CT, USA), and used for further experiments.

### 2.2 SEM and EDX analyses

Scanning electron microscopy (SEM) coupled with energy-dispersive X-ray spectroscopy (EDX) was performed to confirm the synthesis of MnO and ZnO MPs. The MnO and ZnO MPs were placed on a double-faced conductive carbon tape and sputter-coated with platinum. SEM and EDX analyses were performed using JEOL JSM-6510LA (Laboratory of XPS Analysis, Joint Facilities, Faculty of Engineering, Hokkaido University).

### 2.3 Duckweed and the growth condition

The growth of sterilized duckweed, L. *minor/japonica* RDSC ID 5512 (18), was tested for 10 days in a six-well plate (Falcon #353,046, Corning Inc., AZ, USA) containing 9 mL of modified Hoagland medium (mH) in each well by inoculating two fronds. The recipe for mH was as follows: 0.36 mM KNO_3_, 1.68 mM K_2_SO_4_, 0.99 mM CaCl_2_·2H_2_O, 0.42 mM MgSO_4_·7H_2_O, 0.03 mM NaH_2_PO_4_·2H_2_O, 0.012 mM FeSO_4_·7H_2_O, 0.02 mM H_3_BO_3_, 0.002 mM MnCl_2_·4H_2_O, 0.0003 mM ZnSO_4_·7H_2_O, 0.0001 mM CuSO_4_·5H_2_O, and 0.001 mM H_2_MoO_4_ [18]. The pH was adjusted to 7.0 with 0.2 N NaOH. The growth conditions for L. *minor* were: 28 °C, 75 μmol m^–2^ s^–1^ PPFD with a 16 h-photoperiod. Duckweed growth was evaluated by measuring the number of fronds (leaf-like structures) and dry weight after 10 d in culture. Local *Spirodela polyrhiza* HSP001 was also used for starch production under the same growth conditions as described above. Duckweeds were pre-cultured in mH at least for one month to stabilize physiological conditions.

### 2.4 Test design

The experiments were conducted in two steps to evaluate the effects of MnO and ZnO MPs on the duckweed growth. The experimental steps were as follows.

#### 2.4.1 Preliminary growth test of L. *minor* upon exposure to MnO and ZnO MPs

The first experiment was designed to evaluate the effect of treatments on the growth of L. *minor* over a wide range of MnO and ZnO MPs concentrations, using MnSO_4_ and ZnSO_4_ salts as references. The methodology was referred to multi-well culture plates treatments [19]. Duckweeds were grown for 10 days in the presence of MPs and reference salts at the following concentrations: 1, 8, 40, and 1000 mg/L.

#### 2.4.2 Narrowing down beneficial MnO and ZnO MPs concentration

The effective concentration ranges of MnO and ZnO MPs for duckweed growth promotion were examined in the second experiment. This phase involved testing lower concentrations of MnO MPs at 1, 4, 8, and 12 mg/L and ZnO MPs at 1, 2, 3, and 4 mg/L. The experimental setup and conditions were consistent with those used in the first experiment.

### 2.5 Starch content analysis

In the third experiment, a lower concentration of MnO MPs at 1 mg/L was tested on L. *minor* ID 5512 and *S. polyrhiza* HSP001 to assess their effects on starch content as a bioresource value indicator. *S. polyrhiza* was used in this study because of its excellent capacity to produce starch from duckweed [14]. Starch content was measured using rapid total starch (RTS)-NaOH hydrolysis procedure followed by Total Starch Assay Kit (Megazyme, Neogen Japan K.K.). RTS-NaOH hydrolysis was performed by suspending 25–100 mg dry duckweed in 0.2 mL 80 % ethanol, added 2 mL of cold 1.7 M NaOH to vortex every 2 min for 15 min on ice, and stopped reaction by adding 8 mL 600 mM sodium acetate buffer (pH 3.8) containing 5 mM CaCl_2_.

### 2.6 Statistical analysis

Data were analyzed using a *t*-test against no MPs treatment (0 mg/L). Each experiment was performed in triplicate (*N* = 3) and values are expressed as mean ± SD.

## 3 Results

### 3.1 Physicochemical analyses of biogenic MnO and ZnO MPs by SEM and EDX

**Fig. 1:**
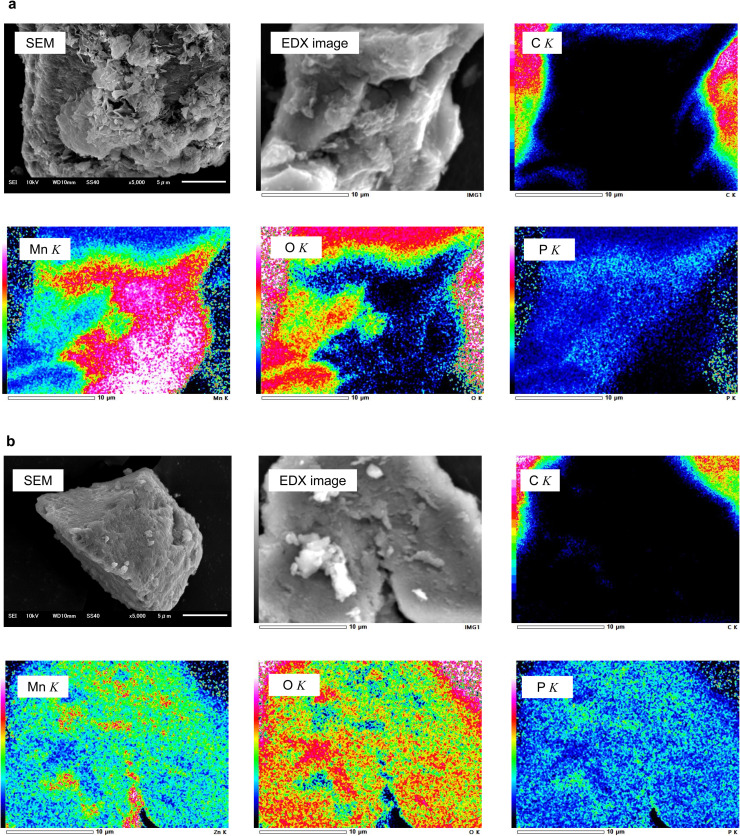
SEM and EDX analyses of biologically synthesized MnO MPs (**a**) and ZnO MPs (**b**). The intensity of X-rays from *K*-shell electrons is shown as a heatmap for each element, C, Mn, O, and P. The high intensity in C *K* and O *K* is due to the carbon tape used for fixing the MPs on the stage. Large MPs (several μm in size) are shown here.

SEM observation revealed that both MnO and ZnO MPs were well dispersed and spherical in shape. EDX quantitative analysis using the ZAF method revealed that the MnO MPs were composed of manganese (63.08 %), oxygen (23.07 %), and phosphorus (13.85 %), with no carbon or organic compounds (Fig. 1a, Supplemental data). In contrast, the ZnO MPs were composed of zinc (49.62 %), oxygen (31.24 %), and phosphorus (18.12 %) with no carbon (Fig. 1b, Supplemental data).

### 3.2 Preliminary growth test of L. *minor* upon exposure to MnO and ZnO MPs

**Fig. 2:**
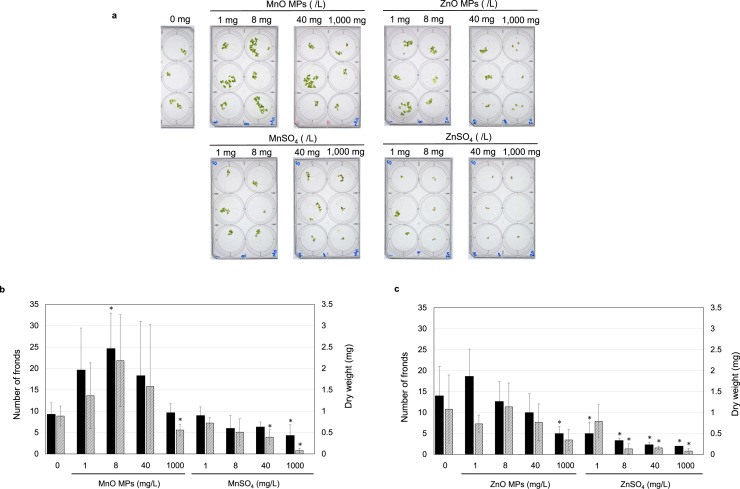
**a.** L. *minor* fronds after 10 days mH culture containing MnO MPs, ZnO MPs, MnSO_4_, and MnSO_4_ at indicated concentrations. **b.** Number of fronds (closed bars) and dry weight (shaded bars) after 10 days in either MnO MPs or MnSO_4_ culture. Initial number of fronds on day 1 was two. **c.** Number of fronds (closed bars) and dry weight (shaded bars) after 10 days in either in ZnO MPs and ZnSO_4_. Initial number of fronds on day 1 was two. *N* = 3. Statistically significant results at *p* value < 0.05 against 0 mg/L are shown as *.

The effects of MnO and ZnO MPs on the growth of L. *minor* were evaluated based on the number of fronds and dry weight after 10 d (Fig. 2). It was observed that MnO MPs at concentrations in the range of 1–40 mg/L and ZnO MPs at 1 mg/L enhanced the duckweed growth. In contrast, MPs at the high concentration of 1000 mg/L negatively affected the growth. On the other hand, MnSO_4_ and ZnSO_4_ in solution generally exhibited inhibitory effects of the duckweed growth.

### 3.3 Narrowing down beneficial MnO and ZnO concentrations

**Fig. 3:**
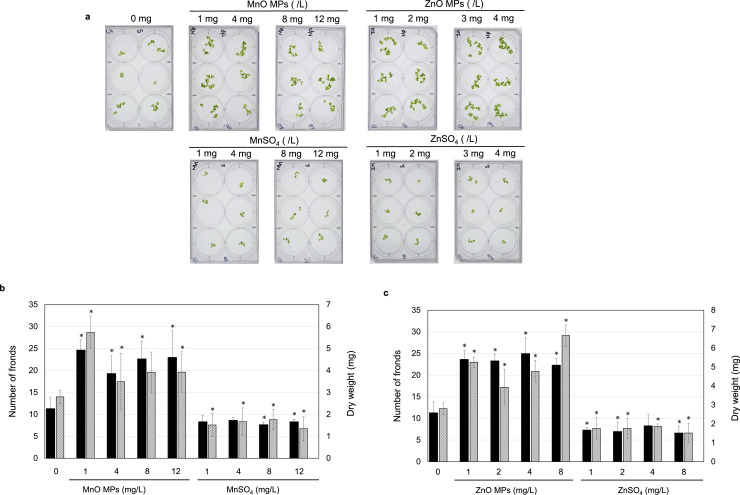
**a.** Observation of L. *minor* fronds after 10 days in culture with MnO MPs and MnSO_4_ at indicated concentrations. Initial number of fronds on day 1 was two. **b.** Number of fronds (black bars) and dry weight (shaded bars) after 10 days in culture with MnO MPs and MnSO_4_. **c.** Number of fronds (closed bars) and dry weight (shaded bars) after 10 days in culture with ZnO MPs and ZnSO_4_. *N* = 3.

In order to verify the positive effect of MPs, L. *minor* was grown near previously observed beneficial concentrations of each MnO or ZnO MPs. The MnO MPs were tested at concentrations of 1, 4, 8, and 12 mg/L, whereas the ZnO MPs were tested at concentrations of 1, 2, 3, and 4 mg/L. In all treatments with MnO and ZnO MPs in this range, the growth of L. *minor* was clearly increased compared to that of untreated L. *minor*. In contrast, MnSO_4_ and ZnSO_4_ solutions slightly decreased the growth of L. *minor* at almost all concentrations (Fig. 3).

### 3.4 Evaluation of starch content in the duckweed after growing with 1 mg/L MnO MPs

Duckweed plant grows two-dimensionally on water surfaces, so it does not need to stand against gravity. This unique reproductive characteristic is accompanied by the chemical composition of the plant body, which is different from that of most plants that grow upright against gravity in soil. The cell wall of duckweed consists of 20.1 % pectin and glucomannan, 35.2 % hemicellulose, 30 % cellulose, and only 5 % lignin [20]. Furthermore, duckweed, which belongs to the Lemnaceae family, is closely related to the Araceae family and has an excellent ability to accumulate starch, especially *S. polyrhiza*. These characteristics make duckweed a favorable biomass resource for biofuel production [14,21]. We further investigated the effect of MPs on duckweed starch content in L. *minor* and *S. polyrhiza* both grown with 0 and 1 mg/L MnO MPs for 10 days*.* It was found that starch contents of L. *minor* and *S. polyrhiza* tended to increase by MnO MPs from 8.3 % and 25.0 % (0 mg/L MnO) to 11.4 % and 41.8 % (1 mg/L MnO) per dry weight, respectively (Table 1).

**Table 1:** Starch contents ( %) in L. *minor* and *S. polyrhiza* (*N* = 3) grown with and without MnO MPs.

**L. *minor***	1	2	3	Average	SD
0 mg/L MnO MPs	2.4	11.2	11.4	8.3	5.1
1 mg/L MnO MPs	10.8	8.8	14.6	11.4	2.9
***S. polyrhiza***	1	2	3	Average	SD
0 mg/L MnO MPs	14.3	18.7	42.1	25.0	14.9
1 mg/L MnO MPs	33.7	73.0	18.7	41.8	28.0

## 4 Discussion

Most reports on the effects of NPs/MPs on duckweed have focused on plant toxicity, including those of Ag, CeO, CuO, Fe_2_O_3_, and TiO_2_ [[22], [23], [24], [25]]. This is because duckweed has been designated as an environmental indicator plant for the evaluation of toxic compounds [26]. For example, ZnO NPs with a size of 12–15 nm showed 12 % and 47 % inhibition of specific growth rate at 0.2 and 4 mg/L, respectively [19]. Reactive oxygen species, such as hydrogen peroxide and hydroxyl radicals, accumulate in plant tissues upon exposure to CuO NPs [27]. On the other hand, it has also been reported that ZnO NPs reduced Cd toxicity in L. *minor* and this protective effect was attributed to the partial adsorption of Cd by ZnO NPs [28]. In this study, we used relatively large MPs (approximately 100 nm or more), which are easily collected by popular centrifugation. This may have revealed the positive effects of MnO and ZnO particles on duckweed. It should be mentioned that CuO NPs inhibited the growth of L. *minor* at 10 mg/L although they formed aggregates larger than 600 nm in the plant medium [29]. It was found that the NPs/MPs produced by biological methods in this study contained almost no organic carbon, but instead contained small but a significant amount of phosphorus. As phosphorus is one of the main essential minerals for plants, the possibility cannot be denied that the phosphorus incorporated into MPs along with the direct effect of MnO and ZnO MPs promoted the growth of duckweed. Duckweed growth promotion technology using plant growth-promoting bacteria have been reported [18]. The protein content of duckweed generally increases in proportion to the increase in growth rate [30]. It is also known that protein and starch contents of the duckweed are inversely correlated [31]. Starch accumulation increases under stressful environments such as high salinity and low nutrition where the growth rate is reduced [32]. It is an interesting observation that the duckweed whose growth was promoted by MnO MPs had an increased starch content. Elucidating the detailed mechanism of the metal oxide MPs affecting duckweed metabolism remains a challenge for the future. In this study, biogenic MnO and ZnO MPs were shown to be potential new fertilizers for enhancing production of high starch content duckweed biomass. It is also noteworthy that as duckweed grows, it absorbs and removes minerals from wastewater of various industries and sewage [14]. MPs should also accelerate light driven ecofriendly water purification coupled with low cost production of raw materials.

## CRediT authorship contribution statement

**Maryam Anar:** Writing – original draft, Visualization, Validation, Methodology, Investigation, Formal analysis, Conceptualization. **Sajjad Kamal Shuvro:** Writing – review & editing, Validation, Resources, Methodology, Investigation, Formal analysis, Data curation. **Muhammad Farooq Hussain Munis:** Writing – review & editing, Supervision, Conceptualization. **Masaaki Morikawa:** Writing – review & editing, Visualization, Validation, Supervision, Project administration, Funding acquisition.

## Declaration of competing interest

The authors declare that they have no competing financial interests or personal relationships that may have influenced the work reported in this study.

## Data Availability

Data will be made available on request.
