# Supplementary material for: Enhancement of the duckweed biomass and starch production utilizing biogenic MnO and ZnO microparticles
Source: Biotechnol Rep (Amst). 2025 Jul 16;47:e00907. doi: 10.1016/j.btre.2025.e00907 (PMC12311953; doi:10.1016/j.btre.2025.e00907)
Supplement: Supplementary file 1 [file mmc1.pdf]

Supplemental Data

Fig. 1s, a

MnO

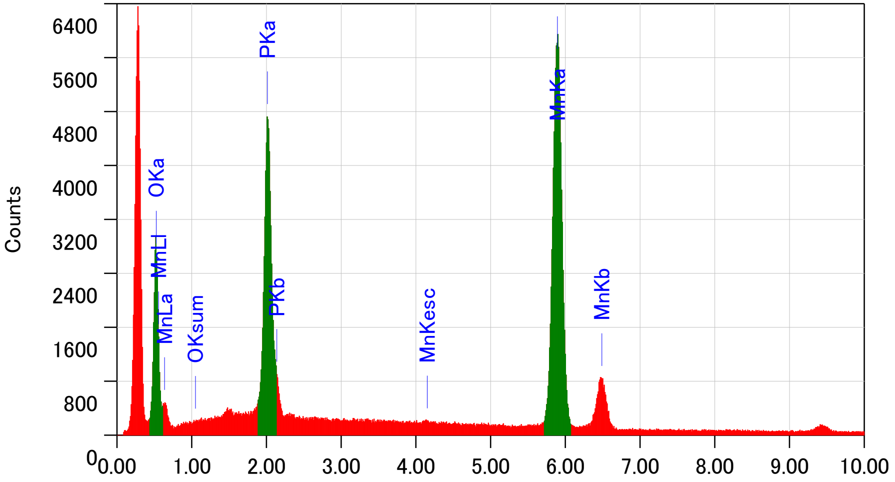

Quantitative analysis by ZAF method

Fitting coefficient value: 0.2047

Element	(keV)	Masst%	σ	Atom%	Cation number
O *K*	0.525	23.07	0.04	47.47	31.4727
P *K*	2.013	13.85	0.02	14.73	12.2473
Mn *K*	5.894	63.08	0.06	37.81	56.2800
Total		100.00			100.00

Fig. 1s, b

ZnO

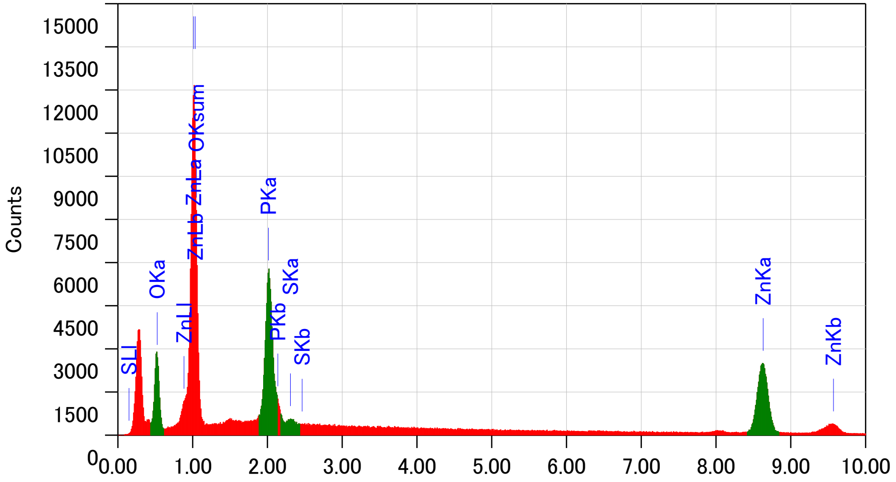

Quantitative analysis by ZAF method

Fitting coefficient value: 0.2047

Element	(keV)	Masst%	σ	Atom%	Cation number
O *K*	0.525	31.24	0.20	58.66	34.6082
P *K*	2.013	18.12	0.11	17.58	15.5755
S *K*	2.307	1.02	0.03	0.96	0.8405
Zn *K*	8.630	49.62	0.33	22.80	48.9758
Total		100.00		100.00	
